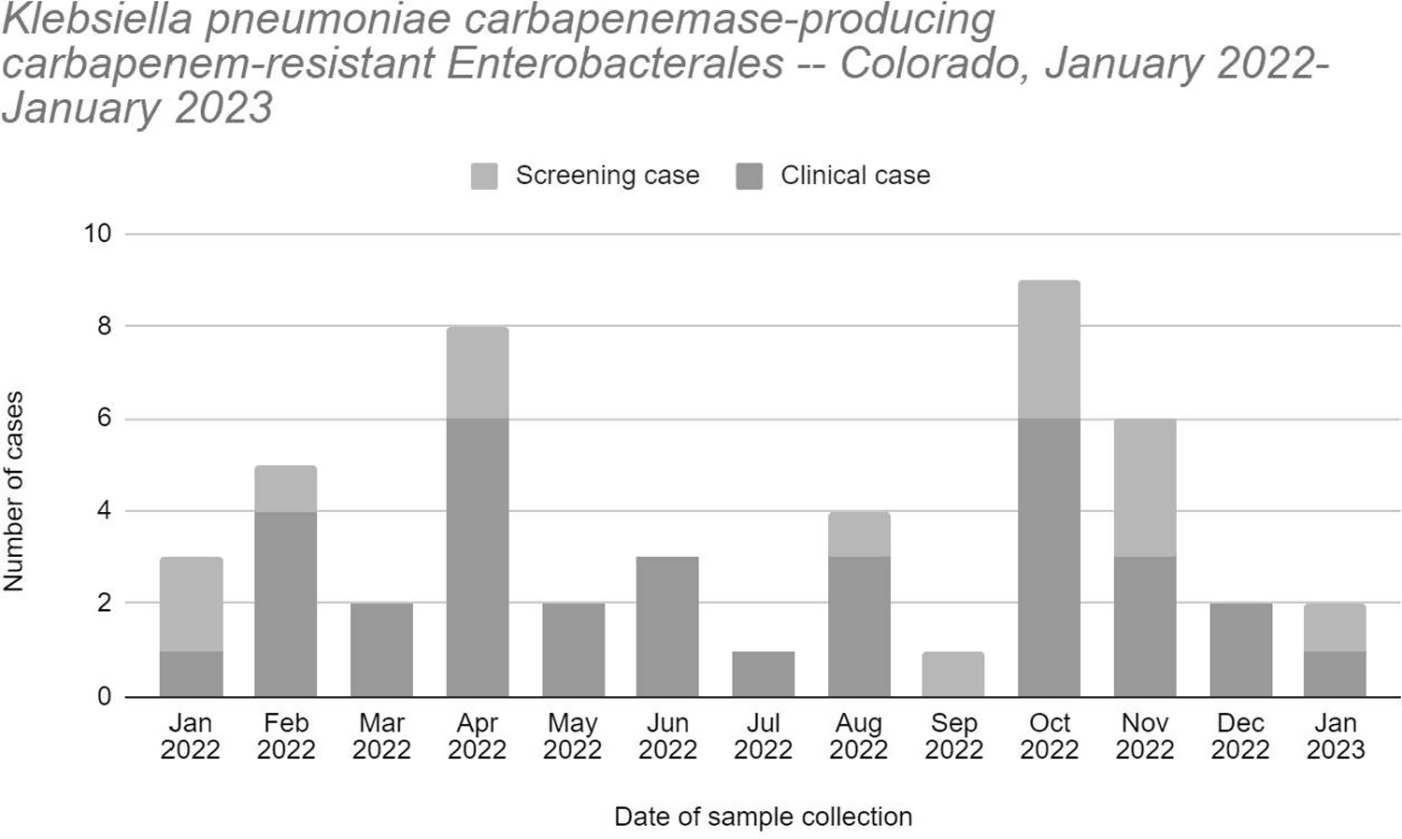# Whole Genome Sequencing to Identify Multiple Clusters of Carbapenemase-Producing Enterobacterales Cases – Colorado, 2022-2023

**DOI:** 10.1017/ash.2024.264

**Published:** 2024-09-16

**Authors:** Jennifer Driscoll, Karlie Hoetzer, Kristen Marshall, Samuel Baird, Helen Johnston, Janell Nichols, Braden Bardach, Marlee Barton, Christopher Czaja, Laura Bankers, Shannon Matzinger

**Affiliations:** Colorado Department of Public Health and Environment; CDC; Colorado Department of Public Health; Ascension All Saints Hospital

## Abstract

**Background:** The Colorado Department of Public Health and Environment (CDPHE) detected an increase in Klebsiella pneumoniae carbapenemase-producing carbapenem-resistant Enterobacterales (KPC-CRE) infections in October 2022. We investigated patient epidemiological links and isolate relatedness to characterize interfacility transmission of KPC-CRE in the Denver metro area and inform regional prevention strategies. **Methods:** We defined a case as polymerase chain reaction detection of KPC from clinical or screening specimens collected during January 2022 – January 2023. Cases were identified through statewide CRE surveillance and carbapenemase testing at the CDPHE laboratory and counted once within a 30-day period. Medical records were reviewed to identify healthcare facility admissions and patient facility overlap in the 12 months prior to sample collection. Whole genome sequencing (WGS) was performed for 34 patients with available KPC-CRE isolates using short- and long-read sequencing techniques. We performed multi-locus sequence typing, generated genome phylogenetic trees, and compared plasmid contig sequences to identify relatedness between KPC-CRE isolates. Clusters were defined as ≥2 genetically related isolates of the same organism or carbapenemase plasmid, from different patients. **Results:** We identified 48 cases (34 clinical and 14 screening) among 39 patients (figure). Patients had a mean age of 52 years (range 16-86) and median of three healthcare facility admissions (range 1-14). Twenty-eight patients (72%) were male. We identified 16 (41%) patients with epidemiological links to one acute care hospital (ACH), 11 (28.2%) patients to one long-term acute care hospital (LTACH), and four (10.2%) patients to each of two ventilator-capable skilled nursing facilities (vSNF). Five distinct clusters of KPC-CRE were identified by WGS among 23 patients (E. hormaechei, two distinct E. cloacae clusters, K. pneumoniae, and K. oxytoca) with linkages to ten healthcare facilities, including two vSNFs, two LTACHs, and six ACHs. Three distinct KPC genes were identified among the clusters: KPC-2, KPC-3, and KPC-4. Genomes assembled from long reads identified identical or similar KPC-gene-containing plasmids across different species or sequence types, suggesting horizontal gene transfer of KPC. **Conclusions:** Multiple KPC-CRE strains co-circulated and were associated with patient movement between acute and post-acute care settings. WGS allowed us to identify multi-facility clusters. Time and location of carbapenemase acquisition were challenging to determine for genetically related isolates when epidemiologic links could not be determined from medical records. This could be due to undetected cases. We notified healthcare facilities of their shared transmission risk and advocated for improved attention to infection control, carbapenemase screening, and communication upon patient transfer.

**Figure:**